# Transcriptomic thermal plasticity underlying gonadal development in a turtle with ZZ/ZW sex chromosomes despite canalized genotypic sex determination

**DOI:** 10.1002/ece3.9854

**Published:** 2023-02-24

**Authors:** Thea B. Gessler, Zhiqiang Wu, Nicole Valenzuela

**Affiliations:** ^1^ Department of Ecology, Evolution, and Organismal Biology Iowa State University Ames Iowa USA; ^2^ Genetics and Genomics Program Iowa State University Ames Iowa USA; ^3^ Guangdong Laboratory for Lingnan Modern Agriculture, Agricultural Genomics Institute at Shenzhen Chinese Academy of Agricultural Sciences Shenzhen China

**Keywords:** eco‐evo‐devo adaptation, embryonic transcriptomes, plasticity versus canalization, sex determination by temperature and sex chromosomes, turtle reptile vertebrates

## Abstract

Understanding genome‐wide responses to environmental conditions during embryogenesis is essential for discerning the evolution of developmental plasticity and canalization, two processes generating phenotypic variation targeted by natural selection. Here, we present the first comparative trajectory analysis of matched transcriptomic developmental time series from two reptiles incubated under identical conditions, a turtle with a ZZ/ZW system of genotypic sex determination (GSD), *Apalone spinifera*, and a turtle with temperature‐dependent sex determination (TSD), *Chrysemys picta*. Results from our genome‐wide, hypervariate gene expression analysis of sexed embryos across five developmental stages revealed that substantial transcriptional plasticity in the developing gonads can persist for >145 Myr, long after the canalization of sex determination via the evolution of sex chromosomes, while some gene‐specific thermal sensitivity drifts or evolves anew. Such standing thermosensitivity represents an underappreciated evolutionary potential harbored by GSD species that may be co‐opted during future adaptive shifts in developmental programing, such as a GSD to TSD reversal, if favored by ecological conditions. Additionally, we identified novel candidate regulators of vertebrate sexual development in GSD reptiles, including sex‐determining candidate genes in a ZZ/ZW turtle.

## Graphical abstract

Trajectory analysis of embryonic, transcriptomic time series data from two species of turtles—one with sex chromosomes and one with temperature‐dependent sex determination revealed transcriptional plasticity in the developing gonads can persist for >145 Myr, long after the evolution of canalized sex determination via sex chromosomes. Lingering thermosensitivity highlights unappreciated evolutionary potential GSD species may harbor for future adaptive shifts in developmental programing, such as a GSD to TSD reversal, if favored by ecological conditions.

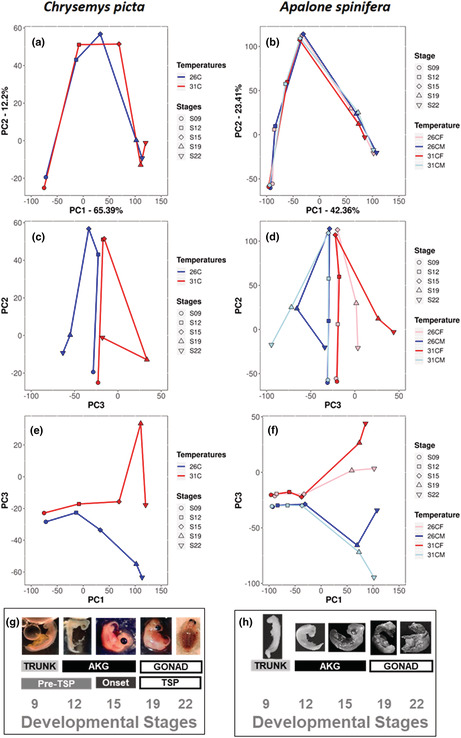

## 1 INTRODUCTION

Understanding the evolution of developmental plasticity and canalization is a fundamental question, as these alternative trajectories generate incredible phenotypic variation targeted by natural selection and driving biodiversity (Beldade et al., 2011; Gomez‐Mestre & Buchholz, 2006; Matesanz et al., 2010). In developmental plasticity, environmental cues shape the phenotypic fate of an individual, rather than genes alone, whereas canalized development is robust to environmental perturbation (West‐Eberhard, 2003). Why some organisms evolve developmental programs susceptible to the whims of the environment remains a fascinating question. Evolutionary theory predicts that when the ecological environment provides reliable cues for phenotypic matching, developmental plasticity increases organismal fitness and is thus adaptive (West‐Eberhard, 2003). Yet, how these external cues are translated into the molecular signals underlying developmental plasticity is not fully known, nor how regulatory networks become insensitive to environmental inputs in canalized systems. This gap hinders our understanding of the evolution of plasticity (or lack thereof) at a molecular level and of the consequences of environmental change.

Sex determination, the commitment to male or female developmental fate, occurs by numerous mechanisms that vary in their level of plasticity (Bachtrog et al., 2014; Bull, 1983; Valenzuela, 2018) and impacts population dynamics by producing initial sex ratios (Abreu‐Grobois et al., 2020; Bull, 1983). Sex‐determining mechanisms (SDMs) span a spectrum from strictly canalized systems of genotypic control (genotypic sex determination – GSD), such as by sex chromosomes, to plastic mechanisms under virtually complete environmental control (ESD), such as temperature‐dependent sex determination (TSD) commonly found in reptiles (Bachtrog et al., 2011; Kratochvíl et al., 2021; Tree of Sex Consortium, 2014).

Turtles are a vertebrate lineage where both GSD and ESD co‐occur (Bachtrog et al., 2014; Stöck et al., 2021) although not within any species studied so far (Mu et al., 2015; Valenzuela et al., 2014). Temperature‐dependent sex determination predominates in turtles and is the likely ancestral state from which GSD evolved multiple times independently as XX/XY or ZZ/ZW systems of sex chromosomes (Bista & Valenzuela, 2020; Janzen & Krenz, 2004; Organ & Janes, 2008; Sabath et al., 2016), which may have been lost in some turtle lineages that reverted back to TSD (Literman et al., 2018; Valenzuela & Adams, 2011). Whether TSD is adaptive in turtles remains the focus of theoretical and empirical research (Schwanz & Georges, 2021; Valenzuela, 2021) as does the evolution of turtle sex chromosomes and their dosage compensation (Bista et al., 2021; Bista & Valenzuela, 2020; Rovatsos & Kratochvíl, 2021). In TSD turtles, the incubation temperatures experienced by the embryo around the middle third of development determine whether the bipotential gonads develop into testes or ovaries by influencing molecular and cellular processes (Merchant‐Larios et al., 2021). This thermosensitive period represents the window of time when the environmental temperatures affect sex ratios the most (Bull & Vogt, 1981), although temperatures before this canonical period can also have an influence (albeit lesser) (Gómez‐Saldarriaga et al., 2016; Valenzuela, 2001). Most turtles develop as males at colder temperatures and as females at warmer temperatures (TSDIa or MF pattern) (Ewert et al., 2004). Thus, turtle sex determination affords an ideal opportunity to study phenotypic plasticity and canalization in an eco‐evo‐devo framework.

Here, we leverage and expand turtle genomic resources to investigate the evolution of the molecular architecture underlying contrasting SDMs, and test for evolutionary shifts in thermosensitivity at the transcriptional level. For this, we compare genome‐wide gene expression during embryogenesis in a GSD and a TSD turtle. Our data permit detangling, for the first time, the effects of sex and temperature on transcription in a turtle with sex chromosomes to illuminate how and why sexual development is plastic in some taxa and canalized in others. Our approach includes RNA‐sequencing from matched samples of *Chrysemys picta* (TSD) and *Apalone spinifera* (GSD), two species from the suborder Cryptodira (referred to by their genus names hereafter). *Apalone* belongs to the family Trionychidae, which lost developmental plasticity for sex determination, replacing it with a ZZ/ZW sex chromosome system ~161–145 mya (timetree.org) (Badenhorst et al., 2013; Literman et al., 2018; Rovatsos et al., 2017). *Chrysemys* serves as a proxy for the ancestral plastic sex determination (Bista et al., 2021). Despite the loss of thermal influence on sex ratio, *A. mutica* and *A. spinifera* exhibit relic and derived thermosensitivity in the expression of some genes (Radhakrishnan et al., 2017, 2018; Valenzuela, 2008a, 2008b; Valenzuela et al., 2013), which could be acted upon by natural selection during future adaptation. Unfortunately, these earlier studies could not disentangle the effects of temperature and sex on gene expression because methods to diagnose the sex of embryos (Literman et al., 2014, 2017) were developed after those studies were conducted. This gap obscures our understanding of the molecular architecture and plasticity underlying both the development of a basic sexual dimorphism (the commitment and differentiation of the gonads) and its evolution with and without sex chromosomes.

To address these questions, we pioneer the application of trajectory analysis (Adams & Collyer, 2009) to a developmental time series of RNA‐seq datasets, to gain insight on global patterns of gene expression that can be elusive otherwise. Specifically, we test how the hyperdimensional path of global gene expression through developmental time differs between colder and warmer incubation temperatures in a TSD turtle (*Chrysemys*), and how this trajectory is influenced by sex, temperature, and their interaction in a GSD turtle (*Apalone*). We then provide a biological interpretation of the plasticity (or lack thereof) of these transcriptional embryonic trajectories.

## 2 MATERIALS AND METHODS

### 2.1 Sample collection

Freshly laid eggs of *Chrysemys* and *Apalone* were collected from turtle farms in Iowa and Oklahoma, respectively, transported to the laboratory soon thereafter, and incubated in the laboratory concurrently and under identical conditions, following standard protocols (Valenzuela, 2009). Briefly, eggs were randomly assigned to incubation boxes and kept at 4% humidity by replacing lost water weekly or before removing eggs. Two constant temperatures were used, 26°C and 31°C, which are within the optimal range for both species and are typically used in studies of their development (Bull & Vogt, 1979, 1981; Gutzke et al., 1987). In *Chrysemys*, 26°C is a male‐producing temperature (MPT) and 31°C is a female‐producing temperature (FPT) (Bull & Vogt, 1981), whereas *Apalone* produces both sexes at these values (Bull & Vogt, 1979) given their ZZ/ZW sex chromosome system (Badenhorst et al., 2013). Embryos of both species were dissected at five identical embryonic stages (Yntema, 1968) to obtain matching samples for comparison. The following tissues were collected for gene expression analysis: trunks at stage 9, adrenal kidney gonad complexes (AKG) at stages 12 and 15, and gonads at stages 19 and 22. In stage 9 embryos, the gonadal primordium cannot be separated in the trunks. In stage 12 embryos, the genital ridge may be present in the AKGs as observed in *Trachemys scripta* turtles (Spotila et al., 1998). And at stage 15, the gonads could not be separated from the AK, lack distinctive internal structure in *Apalone* (Greenbaum & Carr, 2001) and consistently, are bipotential in *Chrysemys* (Bull & Vogt, 1981). In *Chrysemys*, stages 9 and 12 precede the sex‐determining thermosensitive period (TSP), stage 15 lies right before the TSP, while stages 19 and 22 fall within the mid‐ and late‐TSP, respectively (Bull & Vogt, 1981). The sex of *Apalone* embryos was assessed by PCR amplification of sex‐linked markers (Literman et al., 2017), permitting unambiguous diagnosis of individual sex in a simpler manner than by qPCR of rDNA repeats (Literman et al., 2014). *Chrysemys* embryos were presumed to be developing‐males or ‐females if incubated at the MPT or FPT, respectively (Bull & Vogt, 1981). Thus, our sampling permits comparing global gene expression of the same tissues in TSD versus GSD species at equivalent stages of development, corresponding to before and during the thermosensitive period of the TSD taxon. We note that because tissues sampled differ by stage, results through time should be interpreted with caution.

Total RNA was extracted from collected tissues using RNeasy Kits (Qiagen), quantified using a NanoDrop1 ND‐1000 Spectrophotometer, and evaluated for quality by the presence of ribosomal RNA bands in agarose gels stained with ethidium bromide. For *Apalone*, two pools of RNA (biological replicates) per sex were generated per stage and temperature by dividing male or female embryos into two groups of similar or equal size (5–10 embryos per pool). For *Chrysemys*, two pools per temperature per stage were obtained in the same manner using 11–15 embryos per pool. This pooling design captures and accounts for biological variation among individuals which strengthens the differential gene expression analysis. Equal amounts of total RNA per embryo were added to obtain 1 μg RNA per pool, and shipped to Duke Genome Sequencing Core (DUGSIM) for RNA‐seq library preparation and sequencing. The KAPA Stranded mRNA‐seq kit (KK8421) was used on the Sciclone Liquid Handling Workstation to prepare the RNA libraries. RNA libraries were sequenced using Illumina's HiSeq 4000 with 150 bp paired‐end reads. At least 42 M reads were generated per library (average 54 M reads) from which a minimum of 40 M clean reads were obtained (average 52 M reads, or 94%–97% retention rate per library). These correspond to the RNAseq data used in two prior partial analyses of *Dmrt1* splicing and expression in *Chrysemys* (Mizoguchi & Valenzuela, 2020) and of sex chromosome dosage compensation in *Apalone* (Bista et al., 2021). The analyses presented here are distinct from these previous studies and use the full RNAseq datasets for the first time, including a direct between‐species comparison of genome‐wide transcriptional patterns.

### 2.2 Transcriptome assembly

Briefly, FASTQ paired‐end reads were quality trimmed using Trimmomatic (v0.36) (Bolger et al., 2014), concatenated, normalized to a maximum coverage of 30 (Trinity v2.6.6) (Grabherr et al., 2011), and mapped using GSNAP (v20170317) (Wu & Nacu, 2010; Wu & Watanabe, 2005) to the reference *Chrysemys* genome assembly (GCF_000241765.3_Chrysemys_picta_bellii‐3.0.3) (Badenhorst et al., 2015) and to our in‐house *Apalone* genome assembly (BioProject: PRJNA837702). For the alignment of normalized reads, the novel splicing feature was turned on (‐N 1), and a mismatch of seven was allowed (−m 7). This level of mismatch is more conservative than the maximum recommended (−m 10) and resulted in the highest mapping rate. This value was chosen to accommodate the highly heterozygous reads expected from libraries that encompass multiple individuals as described above, which contain a broad representation of the genetic variation present in natural populations. The SAM file of each alignment was converted to a BAM file using Samtools (v1.4) (Li, 2011; Li et al., 2009), and the BAM file was assembled de novo using a genome guided approach as implemented in Trinity (v2.6.6) (Grabherr et al., 2011) with a max intron size of 10,000 and a kmer size of 32. Additional details and parameters used in these steps can be found in the supplementary scripts file.

### 2.3 Generating final reference transcriptome assembly and annotations

Initial transcriptome fragmentation and redundancy were reduced using TransPS (v1.1.0) (Huang et al., 2016; Liu et al., 2014) for transcript‐protein scaffolding on blastx (Camacho et al., 2009) top hits. Prior to running TransPS with default parameters, duplicate copies were removed from the reference proteome using CD‐HIT (v4.6.8) (Fu et al., 2012; Li & Godzik, 2006). Additionally, all blastx results were first reoriented into the sense direction using seqtk (v1.2) (github.com/lh3/seqtk). Top hits from blastx were filtered by best bit score and e‐value and retained for downstream analysis. Transcripts that failed the initial blastx to *Chrysemys* proteins were blasted against chicken (GCF_000002315.6_GRCg6a_protein.faa), the closest vertebrate with the highest quality and well‐annotated genome available. Finally, we used blastn against the RNACentral database (version 11) (The RNAcentral Consortium, 2019) to annotate noncoding RNA (ncRNA) present among orphan transcripts. The final reference assembly was also blasted against the Uniprot vertebrate database (SwissProt v12‐18‐2019) (The UniProt Consortium, 2018) to correct occasional name discrepancies in the *Chrysemys* annotations, primarily for the enrichment analysis and weighted gene co‐expression network analysis (WGCNA) described below. To assess assembly quality before and after scaffolding, BUSCO (v2.0) (Simão et al., 2015) scores were calculated utilizing the Tetrapoda database from Orthodb (v9) (Zdobnov et al., 2016), and read representation in the assembly was determined using GSNAP and Samtools. The SAM file was converted to a BAM file using Samtools, and flagstat was run to determine mapping rates (Table S1). Sequences with >10% Ns were removed from the reference transcriptome prior to differential expression analysis (i.e., 4.2% of reference transcripts for *Chrysemys* and 5.7% for *Apalone*). Additional details are provided in the supplementary scripts file.

### 2.4 Differential expression analysis

Kallisto (v0.46.0) (Bray et al., 2016) was used to obtain counts for the final reference assembly from the trimmed reads which were pseudo‐aligned to the final transcriptome assembly (indexed with a kmer of 31) (see supplementary scripts file for details). Pseudo‐alignment rates (Table S1) were comparable between the raw Trinity assembly and the TransPS assembly. Differential expression analyses were conducted on these counts for each developmental stage separately in the R (v3.5.0) (R Core Development Team, 2018) package DESeq2 (v1.20.0) (Love et al., 2014) using a custom script. Differential expression was calculated between temperatures (26°C vs 31°C) for *Chrysemys*. For *Apalone*, differential expression was modeled at each stage using a full factorial analysis (Y ~ temp × sex) to evaluate differential expression between 26°C and 31°C (temperature effect), between males and females (sex effect), and the temperature‐by‐sex interaction. Because the interaction was nonsignificant for embryonic stages 9, 12, and 15 for most genes, a reduced model (Y ~ temp + sex) was applied to evaluate only temperature and sex effects at those stages. All differentially expressed genes (DEGs) were identified using a corrected *p*‐value <.05.

The transcriptomes of *Chrysemys* were validated by comparing TPM values to previously published qPCR expression data (Mizoguchi & Valenzuela, 2020; Valenzuela et al., 2013) and to previously published transcriptomes (Mizoguchi & Valenzuela, 2020; Radhakrishnan et al., 2017), to assess the similarity of expression patterns for six genes of interest at identical incubation conditions [*Aromatase* (*Cyp19a*), *Sf1* (*Nr5a1*), *Wt1*, *Dax1* (*Nr0b1*), *Sox9*, and *Dmrt1*] across the five embryonic stages, and to other genes from previous studies that used different RNAseq data (Radhakrishnan et al., 2017, 2018). For *Apalone*, validation was conducted to assess the similarity of expression patterns of these genes across development, by comparing TPM values of the same six genes of interest between temperatures only (modeled as Y ~ temp) to the previously published transcriptomic studies that only examined thermal effects on gene expression (Radhakrishnan et al., 2017, 2018).

We analyzed patterns of overlap of DEGs using the R package VennDiagram (v1.6.20) (Chen, 2018). For *Chrysemys*, we identified DEGs with consistent patterns across embryonic stages, whereas for *Apalone*, we identified DEGs by temperature and by sex. Then, we compared a set of reciprocal best blast hits between *Chrysemys* to *Apalone* to identify DEGs with consistent patterns between species at each stage, and genes with sexually dimorphic expression in *Apalone* that may or may not be thermosensitive in *Chrysemys*, to uncover instances of evolutionary retention, gain, or loss of thermosensitivity. Based on these results, we built working hypotheses of potential gene candidates important for sex determination in *Apalone*, and especially those that might contribute to the transition from TSD to GSD in the softshell turtle lineage.

### 2.5 Trajectory analysis

**FIGURE 1:**
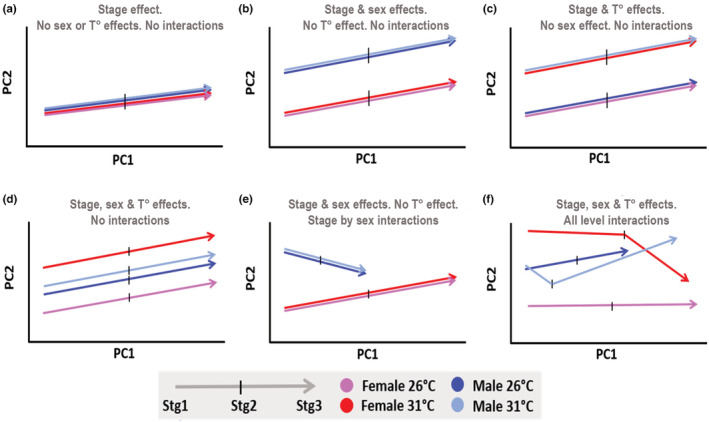
Hypothetical potential results from the trajectory analysis. Panels illustrate a few simplified examples of trajectories over three developmental stages of male and female embryos of a GSD species incubated at two temperatures. All trajectories in (a–d) are of equal magnitude, angle, and shape. Male and female trajectories in (e) differ in both magnitude and angle. Trajectories in panel f differ in magnitude, angle, and shape. Other examples can be found in (Collyer & Adams, 2007).

Gene expression profiles are highly multivariate data, composed of the expression values for all genes per pool of individuals per treatment through time (Adams & Collyer, 2009; Collyer & Adams, 2013), and can be thought of as a temporal trajectory of gene expression (phenotypic response values) over the five embryonic stages. This approach permits the analysis of hyperdimensional genome‐wide expression trajectories, whereas alternative existing approaches address gene‐by‐gene patterns in RNAseq time series (Oh & Li, 2021). First, an ANOVA was conducted to investigate how the genome‐wide transcriptomic response (means of the principal components of gene expression for all replicate RNA‐seq libraries) differed between temperatures in *Chrysemys*, and how they differed in *Apalone* by temperature, sex, and their interaction. A significant interaction term (*p*‐value <.05) suggested the presence of biological interactions for which trajectory analysis is useful to interpret. For this, the resulting multivariate trajectories of gene expression were analyzed for differences in their attributes of magnitude (distance), direction (angle), and shape in the multidimensional gene expression space (Adams & Collyer, 2009; Collyer & Adams, 2013; Figure 1). Although we applied this approach to qPCR data for six genes of interest previously (Valenzuela, 2010), to our knowledge, this is the first application of trajectory analysis at the global transcriptome‐level for developmental time series data. Following procedures outlined by Adams and Collyer (2009), embryonic trajectories of gene expression for both *Chrysemys* and *Apalone* were assessed using the R (v3.5.0) package RRPP (v0.4.2) (Collyer & Adams, 2018). When interpreting these results, we kept in mind that different tissues were analyzed at different stages of development, such that some changes in the trajectories may be attributable to tissue differences. Additionally, as trajectories are hyperdimensional, visual projections in lower dimensions were interpreted with caution, and always corroborated by other data.

Briefly, for the trajectory analysis here, trimmed mean of M values (TMM) normalization (Robinson & Oshlack, 2010) was applied using Trinity to render expression values comparable across libraries. Gene expression values were then log_2_ transformed to correct for heteroskedasticity, after adding 0.0001 to all counts to avoid dividing by zero in downstream calculations, and the positive effect of this correction was visualized by comparing the results from a principal components analysis (PCA) performed before and after the transformation (results not shown). Data were fit to a general linear model (*Chrysemys*: gene_expression ~ temperature*stage; *Apalone*: gene_expression~ temperature‐sex*stage) using lm.rrpp(), and significance assessed by ANOVA. Trajectory analysis was run in RRPP using trajectory.analysis(), and trajectories were evaluated for differences in magnitude, direction, and shape, using a Bonferroni‐corrected alpha (applied with p.adjust()) when multiple hypotheses were tested on the same dataset (which was the case for *Apalone*).

### 2.6 Enrichment analysis

Mapping to the UniProt database (described above) was performed prior to enrichment analysis. For *Chrysemys*, 25,527 of 29,607 gene models in the reference transcriptome had a hit in SwissProt, and 18,084 had a GO ID associated with that annotation. For *Apalone*, 23,246 of 25,696 gene models in the reference transcriptome had a hit in SwissProt, and 16,849 had a GO ID associated with that annotation. Higher gene model numbers in *Chrysemys* and *Apalone* are due to the annotation of isoforms present in the *Chrysemys* and/or chicken protein sequences used for assembly redundancy reduction and annotation.

Enrichment analyses were run using Ontologizer (v2.1) (Bauer et al., 2008). Model‐based gene set analysis (MGSA) was used as the calculation method (Bauer et al., 2010), ignoring genes without associations between genes and GO Terms (option ‐i). Gene Ontology files required to run the enrichment analysis were downloaded from geneontology.org on 01/08/2020 (Ashburner et al., 2000; The Gene Ontology Consortium, 2019). For *Chrysemys*, the enrichment was calculated for DEGs that were upregulated at MPT [26°C‐biased] and at FPT [31°C‐biased]. For *Apalone*, enrichment was calculated for DEGs that were male‐biased at 26°C, male‐biased at 31°C, female‐biased at 26°C, female‐biased at 31°C, and alternatively, for genes that were 26°C‐biased in males, 26°C‐biased in females, 31°C‐biased in males, 31°C‐biased in females, using the same log_2_(FoldChange) relationship as for *Chrysemys*.

### 2.7 Weighted gene correlation network analysis—WGCNA

To test for the presence of modules in the gene regulatory network of sexual development, we employed the WGCNA R package (v1.69) (Langfelder & Horvath, 2008; Zhang & Horvath, 2005) to construct (i) a turtle consensus network, (ii) a *Chrysemys*‐specific network, and (iii) an *Apalone*‐specific network from reciprocal best blast hits between the two transcriptomes. To construct the modules, we followed guidance provided by the online tutorials (https://horvath.genetics.ucla.edu/html/CoexpressionNetwork/Rpackages/WGCNA/Tutorials/). Counts were rounded to integers, filtered to a minimum cross‐library read count of at least 20, and transformed using varianceStabilizingTransformation() from the DESeq2 package. Data were then cleaned and clustered, and a soft‐power of 8 was selected as it best met the assumption of a scale‐free topology. Then, consensus and species modules were built.

Weighted gene correlation network analysis uses (1) correlation to measure co‐expression and thus, interaction among genes; (2) hierarchical clustering to identify co‐expression modules (highly correlated groups of genes); and (3) eigengene network analysis to define module relationships (Langfelder & Horvath, 2008). Briefly, network nodes are gene expression profiles, edges between genes are the pairwise correlations between their gene expression, and connectivity is how highly co‐expressed a gene is relative to other genes in a module (Langfelder & Horvath, 2008). A module's eigengene is its principal component, or representative (weighted average) gene expression profile. When interpreting gene co‐expression data, we are mindful that these species‐specific networks are based on highly heterogeneous data that vary by sex, temperature, and embryonic stage/tissue, which could influence the construction of networks.

Overlap between consensus and species‐specific modules (and the genes involved) were calculated, as well as the network adjacency (i.e., connection strength between nodes) and preservation (i.e., conservation or similarity) among species‐specific modules. Given the high conservation of the vertebrate sex determination network (Merchant‐Larios et al., 2021; Morrish & Sinclair, 2002), we predicted that high module overlap would exist, but also that some differences in the molecular circuitry of TSD and GSD mechanism would be present between *Chrysemys* and *Apalone*.

## 3 RESULTS

### 3.1 Transcriptome assembly and validation

A large fraction of reads mapped to the reference genomes: 94.27% for *Chrysemys* and 86.18% for *Apalone*. Raw Trinity assemblies contained >1 M transcripts with high redundancy. These were reduced to 29,607 gene models for *Chrysemys* and 25,696 for *Apalone* using TransPS with minimal loss of data pre‐ and postscaffolding, less duplication, and greater completeness based on BUSCO scores (Table S1). Furthermore, pseudocounts from Kallisto were only slightly lower after running TransPS indicating that a similar amount of data was utilized in both cases (Table S1). The final transcriptome for each species had BUSCO scores of 93.90% and 91.50% for complete transcripts, respectively (Table S1). Validation of the resulting reference transcriptomes using six genes of interest [*Wt1*, *Sf1* (*Nr5a1*), *Dax1* (*Nr0b1*), *Sox9*, *Aromatase* (*Cyp19a1*), *Dmrt1*] profiled earlier (Mizoguchi & Valenzuela, 2020; Radhakrishnan et al., 2017; Valenzuela et al., 2013) revealed similar expression profiles from all datasets (Figure 5), with only two exceptions detected for *Chrysemys* between transcriptomic studies [(Radhakrishnan et al., 2017) and this study]. Similarly, using the simpler model (Y ~ temp) in *Apalone* that disregarded sex information revealed similar results for the same six genes to previous analyses using unsexed embryos (Table S19) for stages 9–15, but differences for stages 19 and 22 were observed. Namely, the simple model detected no differential expression by temperature in stage 19 and 22 gonads, likely because differences due to sex and temperature were confounded and canceled each other out in the previous study (Radhakrishnan et al., 2017). Thus, sexing *Apalone* embryos and using more embryos per library here, provided greater sensitivity to detect differential expression of gene regulators of vertebrate gonadogenesis than before (Radhakrishnan et al., 2017).

#### 3.1.1 Wilms tumor 1

Because the *Wt1* genomic sequence is split between two scaffolds in the *Chrysemys* reference genome, we validated *Wt1* expression patterns using the *Trachemys* genome as a reference (GCF_013100865.1_CAS_Tse_1.0_genomic.fna). In *Trachemys*, *Wt1* contains three annotated isoforms X1, X2, and X3, which correspond to a sequence with KTS (+KTS), a sequence without KTS (‐KTS), and a short sequence containing KTS (+KTS‐short). Reads that had mapped to *Chrysemys'* two *Wt1* fragments and to the scaffold containing *Wt1* in *Apalone* were extracted and analyzed using the same pipeline described for our main analysis. Results using *Trachemys* as reference corroborated our main results overall. Namely, both *Chrysemys* and *Apalone* expressed primarily two *Wt1* isoforms, +KTS and +KTS‐short, but *Chrysemys* favored +KTS, whereas *Apalone* favored +KTS‐short. This pattern agrees with our original result showing expression of two *Wt1* isoforms, one more highly expressed in *Chrysemys* and the other in *Apalone*.

### 3.2 ANOVA and trajectory analysis uncover transcriptional dimorphism and thermal plasticity in *Apalone*

Given the complex and multivariate nature of transcriptomic data, trajectory analysis was used to understand the significant interaction terms in the genome‐wide transcriptional responses identified via ANOVA (Table 1—All Genes). For *Chrysemys*, we found that developmental stage, temperature, and their interaction all had a significant effect on the expression of DEGs, at a Benjamini–Hochberg corrected α < 0.05, whereas only stage was significant when genome‐wide transcription was included, likely because subtler signals in DEGs were masked by the noise from genes with monomorphic expression (Table 1—DE Genes). In *Apalone*, data were divided into sex‐by‐temperature subsets (26°C‐female, 31°C‐female, 26°C‐male, 31°C‐male) and all ANOVA terms were significant, both genome‐wide and DEGs only (Table 1). Below, we compare trajectories between species for the DEGs.

**TABLE 1:** Results of ANOVA of gene expression in *Chrysemys picta* and *Apalone spinifera* genome‐wide or for DEGs only.

Gene set	Factor	Z score	*p*‐value
*Chrysemys picta*			
All Genes: Stages 9–22	Temperature	1.3167	.1030
	Stage	5.5806	***.0005***
	Interaction	0.3574	.3950
DE Genes: Stages 9–22	Temperature	2.1528	***.0265***
	Stage	5.4237	***.0005***
	Interaction	3.6743	***.0005***
*Apalone spinifera*			
All Genes: Stages 9–22	TempSex	1.8544	***.0455***
	Stage	9.8548	***.0005***
	Interaction	2.2748	***.0075***
DE Genes: Stages 9–22	TempSex	3.2104	***.001***
	Stage	8.7623	***.0005***
	Interaction	6.4318	***.0005***

*Note*: For *Chrysemys*, the model tested the effect of temperature, stage, and their interaction, whereas for *Apalone* the model tested the effect of sex‐by‐temperature, stage, and their interaction. Significant *p*‐values are denoted in bold italics.

**FIGURE 2:**
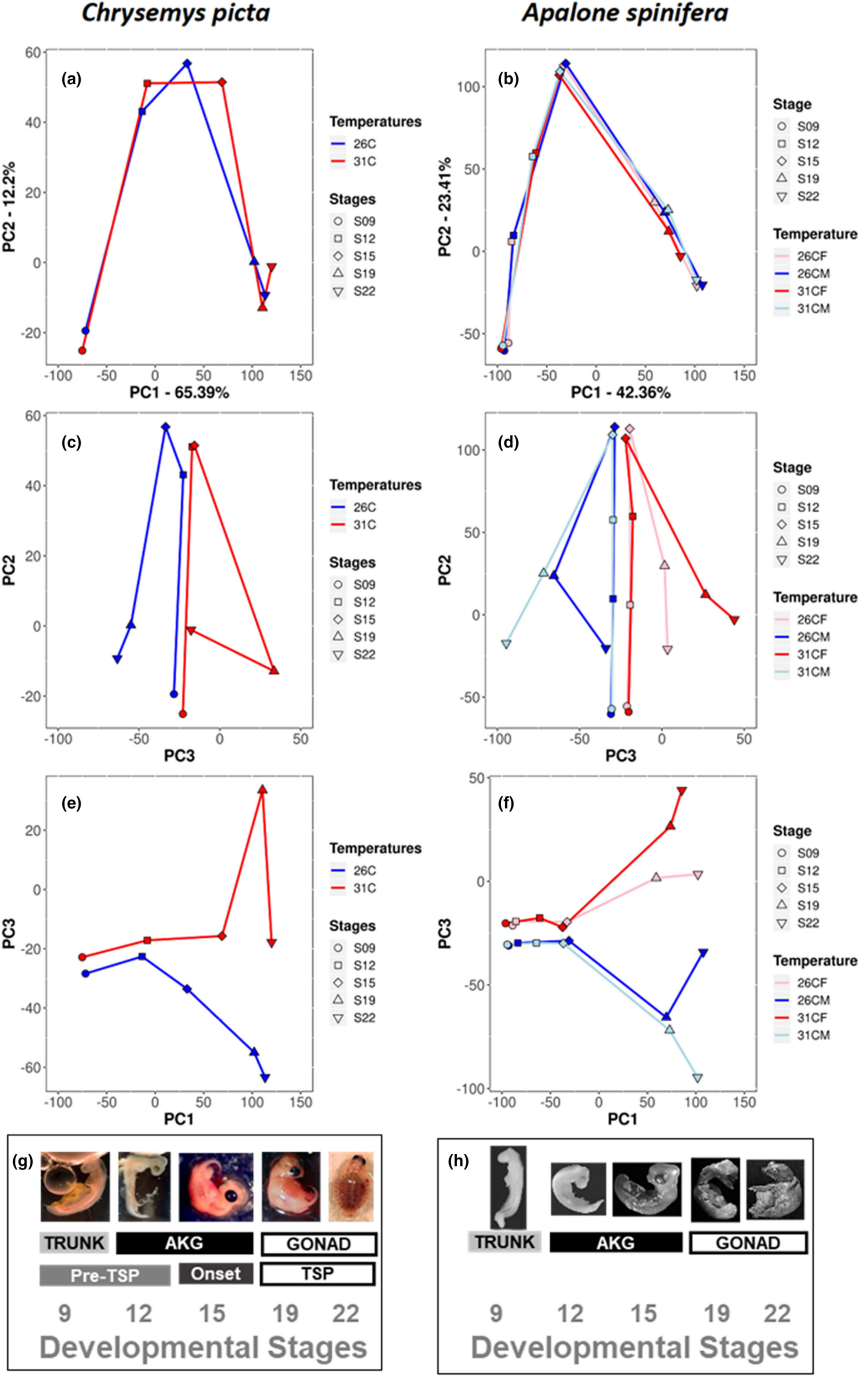
Principal component plots of multivariate gene expression trajectories of *Chrysemys picta* and *Apalone spinifera*. Principal components PC1, PC2, and PC3 are presented from 26°C (blue) and 31°C (red) treatments for *Chrysemys* (left panels a,c,e) and for 26°C‐females (pink), 26°C‐males (blue), 31°C‐females (red), and 31°C‐males (light‐blue) for *Apalone* (right panels b,d,f). Panels illustrate PC1 vs PC2 (a,b), PC2 vs PC3 (c,d), and PC1 vs PC3 (e,f). Embryonic stages sampled are illustrated in panels g and h: *Chrysemys* photos from our lab; softshell photos reproduced from (Tokita & Kuratani, 2001) (stages 9–12 *Pelodiscus sinensis*) and from (Greenbaum & Carr, 2002) (stages 15–22 *Apalone spinifera*) with permission.

For *Chrysemys*, gene expression trajectories differed in magnitude (the amount of change exhibited by DEGs), direction (the sets of DEGs), and shape (changes in magnitude and/or direction through two or more embryonic stages) (Table 2; Figure 2a,c,e). For *Apalone* (Table 2), results from the trajectory analysis across all embryonic stages were consistent with the sex‐by‐temperature interaction identified by ANOVA. Namely, the trajectories across stages (Figure 2b,d,f) exhibited similar magnitude, but the direction of change differed between males and females at 31°C (sex effect), and between temperatures for males (temperature effect), revealing differences in the set of genes that were differentially expressed. Furthermore, temperature affected the shape of the female trajectories under 26°C and 31°C, indicating consecutive changes in magnitude and or direction across two or more embryonic stages. Analyzing *Apalone*'s data with the model used for *Chrysemys* (excluding sex as a factor) revealed that the lack of magnitude differences in *Apalone* was explained by our detangling of the effects of sex and temperature on development in this GSD turtle.

**TABLE 2:** Results of trajectory analysis for *Chrysemys picta* and *Apalone spinifera*.

Stages compared	Attribute of change	Z score	*p*‐value or Bonferroni corrected *p*‐value	Effect type
*Chrysemys picta*				
Stages 9–22	Magnitude	3.446172	***.0025***	
	Direction	4.721982	***.0005***	
	Shape	2.634093	***.0095***	
*Apalone spinifera*				
Stages 9–22	Direction: 26CF:31CM	4.479990	***.006***	Sex and temperature
	Direction: 26CM:31CF	4.403077	***.003***	Sex and temperature
	Direction: 26CM:31CM	3.148147	.054	Temperature
	Direction: 31CF:31CM	5.206589	***.003***	Sex
	Shape: 26CF:31CF	3.525388	***.009***	Temperature
	Shape: 26CF:31CM	2.539313	***.048***	Sex and temperature
	Shape: 26CM:31CF	2.652483	.051	Sex and temperature

*Note*: Results include effect sizes (z scores) and significance of trajectory attributes (magnitude, direction, and shape). For *Apalone* significance was corrected for multiple comparisons, and type of effect detected is presented based on pairwise comparisons. Significant *p*‐values are denoted in bold italics, whereas *p*‐values in regular font are marginally significant (<.055).

The first principal component (PC1) of the trajectory analysis for both species captures mostly developmental time (Figure 2a,b). Plotting PC3 versus PC1 and PC3 versus PC2 for *Chrysemys* reveals divergence by temperature (Figure 2c,d), which is captured by directional differences between trajectories. For stages 9 and 12 (trunks and AKGs, respectively), trajectories are primarily parallel, but by the onset of the TSP (stage 15) sexually dimorphic transcription becomes evident in the AKGs, is accentuated through stage 19 gonads, and lessens by stage 22 in the gonads, the end of *Chrysemys*' TSP (Bull & Vogt, 1981). Genes influencing the trajectories the most are listed in Tables S2 and S3, which for *Chrysemys* included many transcriptional regulators (*Eloa1*, *Tet1*, *Nr5a2*, *Smarca2*, *Tcf7l2*, *Rbfox2*, *and Zic4*), whereas in *Apalone*, fewer genes had higher impact and these included genes associated with development (*Lhx2* and *Dgcr2*).

Our *Apalone* data permitted disentangling temperature effects from sex effects in a GSD turtle for the first time. Indeed, the plots PC3 versus PC1 and PC3 versus PC2 depict sex and temperature effects (Figure 2d,f). Alike *Chrysemys*, stages 9–12 trajectories are mostly parallel in *Apalone*, although a potential temperature effect on magnitude is observed by stage 12. Male and female trajectories re‐converge at stage 15 (although a temperature effect remains evident). Angular variation increases later due to thermal responses within each sex, and both sex and temperature contribute to the divergence of the trajectories at stages 19–22, with sexually dimorphic transcription accentuated under warmer conditions, whereas male and female gene expression is less dimorphic at 26°C. Developing ovaries were more thermally plastic at stages 19–22, whereas testicular transcription was more canalized at stage 19 and more thermosensitive at stage 22. Generally, gene expression differences are most extreme at stage 22 for *Apalone* and at stage 19 for *Chrysemys*.

### 3.3 Enrichment analysis

The enrichment analysis revealed no consistent shared GO terms between species (see Table S4). All cases of enrichment that spanned multiple developmental stages in *Chrysemys* were observed at FPT, and included oxidation–reduction process, among others. Two of eight stage‐spanning cases in *Apalone* were enriched in females and not by temperature, and the other six occurred in males. Interestingly, the terms enriched by temperature in *Apalone* never spanned multiple stages.

### 3.4 Species comparisons reveal candidate sex‐determining genes in *Apalone* with canalized sex‐specific expression in GSD turtles

**FIGURE 3:**
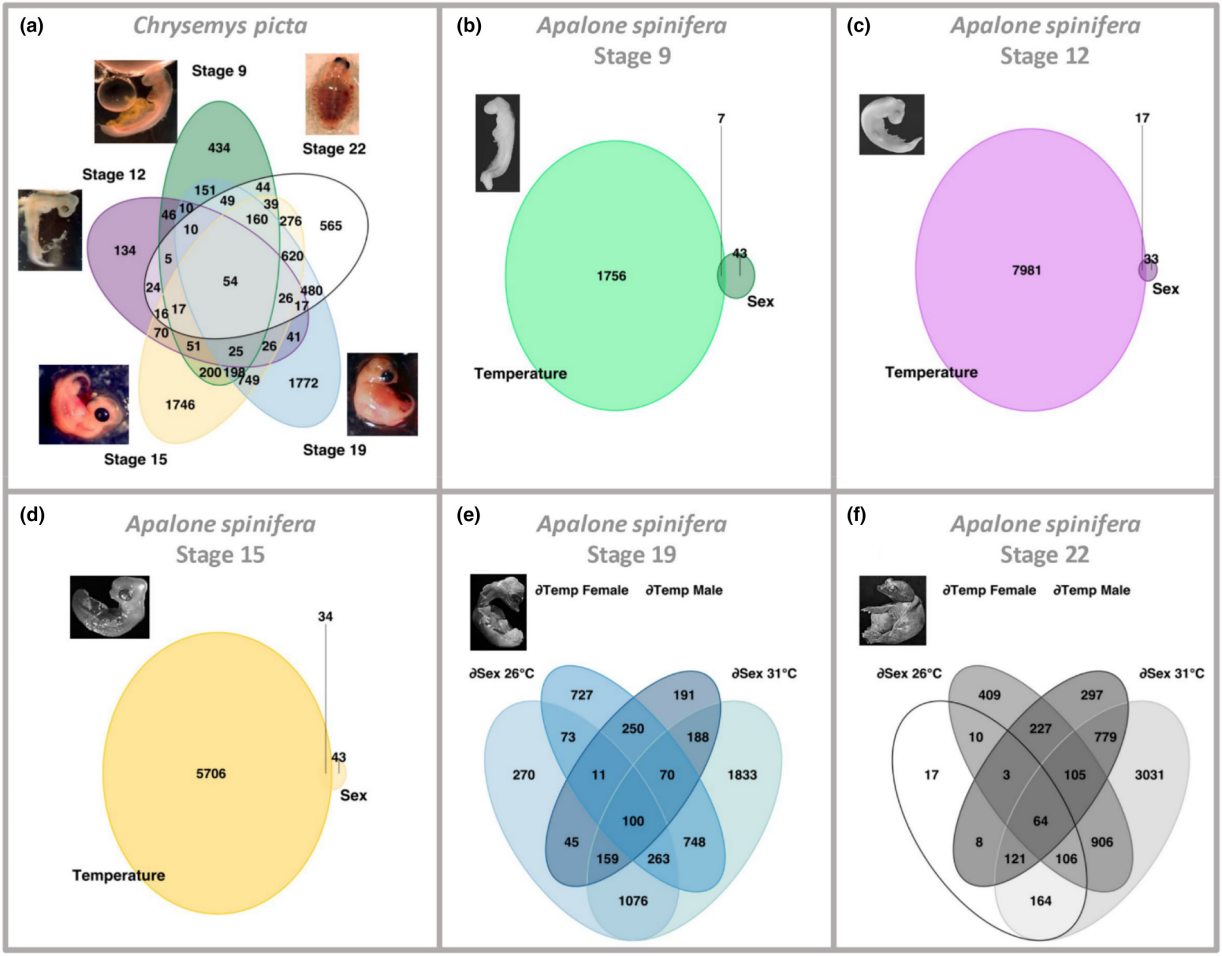
Venn diagrams illustrating the overlap of differentially expressed genes (DEGs) between developmental stages of *Chrysemys picta* and *Apalone spinifera*. DEG overlap across stages for *Chrysemys* (a). DEG overlap for *Apalone* between conditions (sex vs. temperature) at stages 9 (b), 12 (c), 15 (d), 19 (e), and 22 (f). ∂ = between. ∂Sex 26°C = DEGs between males and females incubated at 26°C. ∂Sex 31°C = DEGs between males and females incubated at 31°C. ∂Temp Female = DEGs between 26°C and 31°C in females. ∂Temp Male = DEGs between 26°C and 31°C in males.

Around half of *Chrysemys* DEGs between temperatures occurred at a single developmental stage, mostly in the AKGs at the onset of the TSP (stage 15) and in the gonads at mid‐TSP (stage 19). A larger number of DEGs occurred across multiple stages during the TSP than before (Figure 3a), perhaps because stages 9–15 contained mixed tissues. Full lists of DEGs are presented in the Tables S5 and S6.

In *Apalone*, most DEGs occurred between temperatures and fewer between sexes at stages 9–15, with negligible sex‐by‐temperature interaction (<20 genes per stage), highlighting considerable thermal plasticity (either retained or derived) in the trunks and AKGs of this GSD species (Figure 3b–d). By contrast, genes in gonads at stages 19–22 (which correspond to the mid and late‐TSP of *Chrysemys*) showed a significant sex‐by‐temperature interaction in *Apalone* (360 and 1314 genes, respectively). For full lists of genes, see Table S7 and S8.

**FIGURE 4:**
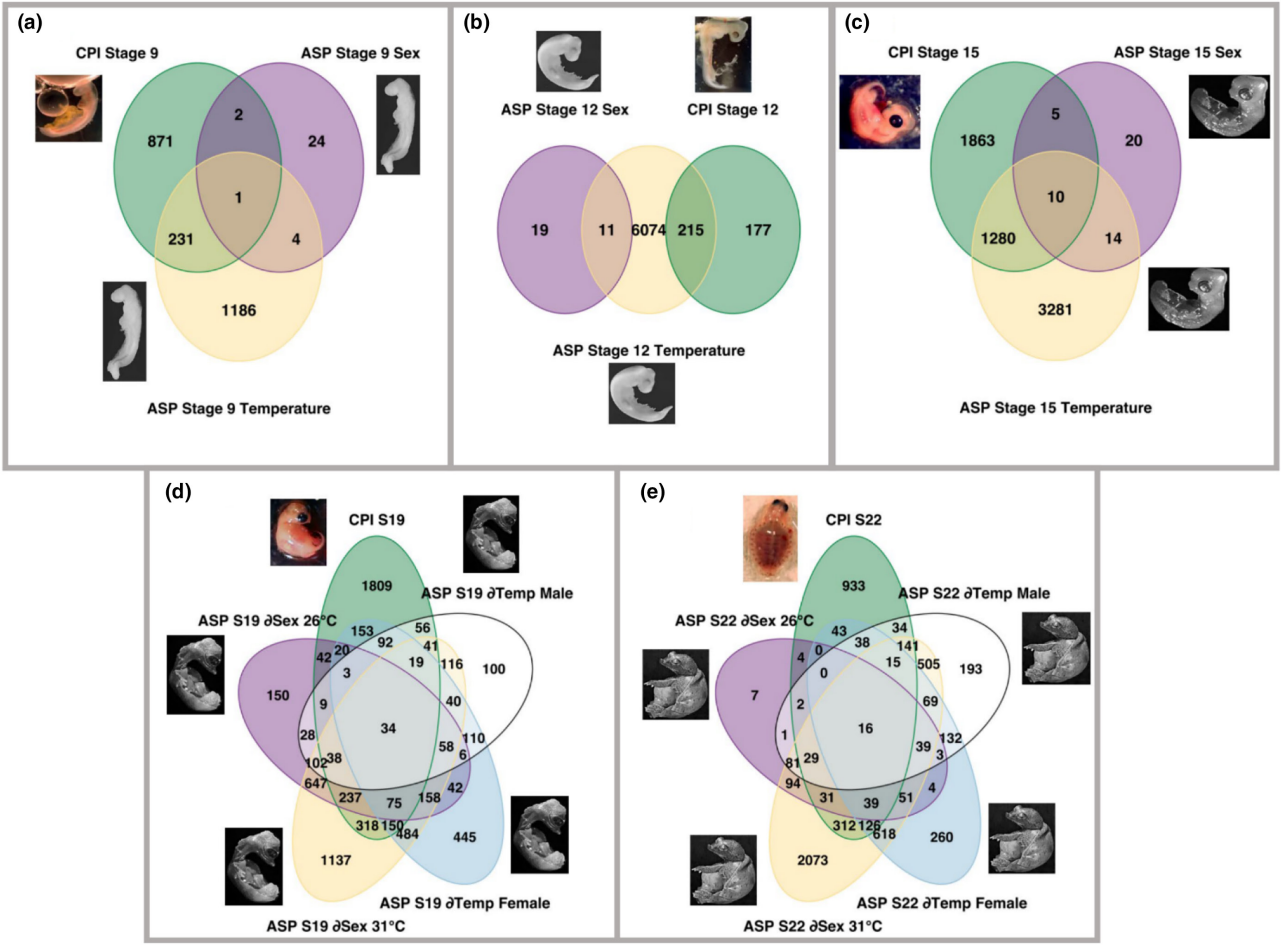
Venn diagrams of the overlap of differentially expressed genes (DEGs) between *Apalone* and *Chrysemys* at stage 9 (a), 12 (b), 15 (c), 19 (d), and 22 (e). *Apalone* data are grouped into DEGs by temperature and DEGs by sex for stages 9–15 (a–c), and by temperature and sex in a full factorial analysis (d and e). ∂ = between. ∂Sex 26°C = DEGs between males and females incubated at 26°C. ∂Sex 31°C = DEGs between males and females incubated at 31°C. ∂Temp Female = DEGs between 26°C and 31°C in females. ∂Temp Male = DEGs between 26°C and 31°C in males.

Cross‐species analysis of DEGs by stage (Figure 4) uncovered numerous genes that are thermosensitive in *Chrysemys* (TSD) but not in *Apalone* (GSD), except at stage 12 (Figure 4b), when DEGs in *Chrysemys* are less abundant relative to other stages. Interestingly, there are also many thermosensitive DEGs unique to *Apalone* (Figure 4), especially at stages 19–22 (Figure 4d,e), yet many were differentially expressed by sex also. For full lists of genes see Tables S9–S13.

Importantly, we identified seven novel candidate sex‐determining genes in *Apalone* which lost thermosensitivity relative to *Chrysemys* and exhibit early sex‐specific expression at stage 9 (*Rrp8*, *Clh1*) and stage 15 (S*26a1*, *Sc5a1*, *Msi1h*, *Sat2*, *Ppil2*). Substantially more genes showed this pattern (loss of thermosensitivity accompanied by sexually dimorphic expression) at later stages in *Apalone* (Stage 19: 597 genes, Stage 22: 347 genes), revealing significant canalization of gonadogenesis at stages equivalent to *Chrysemys* TSP when plastic responses are observed. For full lists of genes, see Tables S9–S13.

Our approach offered greater sensitivity to detect differential expression for known gene regulators of vertebrate gonadogenesis (Figure 6; Table 3). Among these, we note that the testis differentiation gene *Dhh* entirely reverses its expression pattern between the two species. It shows upregulation at 31°C throughout *Chrysemys* TSP, whereas in *Apalone*, *Dhh* is upregulated at 26°C during those same stages but is male‐biased at stages 19–22 (Figure 6). Furthermore, because response to stress may mediate the evolution of ESD in amniotes (Straková et al., 2020), we searched qualitatively for genes annotated as related to stress response (response to cold, response to heat), present in the transcriptomes of *Chrysemys* (175 genes) and *Apalone* (167 genes) (Ashburner et al., 2000; The Gene Ontology Consortium, 2019), and showing consistent pattern across at least three stages. In *Chrysemys*, 17 such stress‐DEGs were detected, including several heat‐shock proteins and *Cirbp* (a TSD candidate gene upregulated in *Chrysemys* across all stages) upregulated at 31°C, and four genes upregulated at 26°C (Table S14). In *Apalone*, only 1 gene (*Atp2a2*, a gene implicated in calcium transport) was male‐upregulated across stages 9–15 (Table S15). For stages 19–22 in *Apalone*, many stress‐related candidates for sexual development (Straková et al., 2020) were detected at 26°C, including *Ano1*, *Atp2a2*, *Ppargc1a*, *Sst*, *Tgfb1i1*, and *Fosl2*. Interestingly, all stress DEGs found in *Chrysemys* were both differentially expressed by sex and by temperature at some stage in *Apalone*.

**TABLE 3:** Novel or confirmed differential expression (or lack thereof) in *Chrysemys picta* and *Apalone spinifera* of previously studied genes of interest.

Taxa	Pattern	Genes
CPI	Confirmed upregulation at 26°C (developing male)	*Amh, Dax1, Dmrt1, Dmrt2, Fog2, Gata4, Kdm3a, Lhx9, Sf1, Sox9, Wt1*
	Confirmed upregulation at 31°C (developing female)	*Aromatase, Ctnnb1, Dhh, Foxl2, Gata2, Lhx1, Sox9, Srd5a2*
	Novel differential expression	*Apc, Cbx2, Ctnnb1, Dhh, Fhl2, Gata2, Gata4, Igf1r, Insr, Kdm3a, Lhx1, Lhx9, Ptch1, Six1, Sox9*
	Monomorphic expression across all stages	*Cbln4, Cyp26b1, Dmrt3, Esr2, Rspo1, Wnt4*
ASP	ZW Female upregulation >1 stage	*Aromatase, Ctnnb1, Emx2, Foxl2, Lhx9, Rspo1, Six4, Wt1*
	ZZ Male upregulation >1 stage	*Amh, Dhh, Dmrt1, Dmrt3, Fgfr2, Fog2, Insr, Ptch1, Sox9*
	Thermosensitive	*Amh, Apc, Ar, Aromatase, Cbx2, Ck1, Ctnnb1, Cyp26b1, Dhh, Dmrt1, Dmrt2, Emx2, Esr2, Fgf9, Fgfr2, Fhl2, Fog2, Foxl2, Gata2, Gata4, Igf1r, Insr, Kdm3a, Lhx9, Ptch1, Rspo1, Sf1, Six1, Six4, Sox9, Wnt4, Wt1*

*Note*: Details in Supplementary excel file.

### 3.5 WGCNA point to part conserved and part evolutionarily labile regulation underlying turtle sexual development

Broad similarities and differences between *Chrysemys* and *Apalone* were detected using WGCNA that help formulate working hypotheses for further studies by identifying groups of highly co‐expressed genes within species (species‐specific modules) and across species (consensus modules) as detailed in the Tables S16–S18. Not surprisingly, species level networks contained fewer and larger modules (*Chrysemys*: 8 modules; *Apalone*: 12 modules) than the consensus network across species (24 modules) (Figure 7a,b). Modular co‐expression preservation (conservation) between species is illustrated in Figure 8. Importantly, the correlation pattern of consensus modules is not always preserved between species, revealing changes in co‐expression patterns of groups of genes across taxa. Indeed, in several cases, consensus modules assigned to a single *Chrysemys* module (Figure 7a) were less correlated within *Apalone* (Figure 7b). Yet, we also found evidence of preservation of CPI‐1 and CPI‐6 modules within *Apalone*, but weak to moderate for CPI‐3, CPI‐4, CPI‐5, and CPI‐7, and none for CPI‐2 and CPI‐8 (note that modules were independently constructed in *Apalone*, such that module number ID do not indicate the same module between taxa). Thus, only some modular structure is preserved between the two species.

We examined the module membership of several genes that are interesting known candidates for a role in sex determination to determine whether any showed similar co‐expression patterns indicative of a cooperative role. We observed that *Wt1*, *Ar*, *Esr1*, and *Kdm6b* all belong to consensus module Cons‐24; *Sox9* and *Dmrt1* belong to Cons‐11; and *Amh* and *Rrp8* belong to Cons‐23, revealing consensus modules with distinct member elements that are conserved across species. By contrast, *Aromatase*, numerous epigenetic regulatory genes such as *Dicer*, *Ago2*, *Dnmt1*, and several histone demethylases belong to Cons‐14, the module with the lowest preservation in co‐expression patterns between species, indicating that the connections of these elements within the sexual development network are evolutionarily labile. Furthermore, *Sf1* and *Trpv4* were not observed in any consensus modules, and their failure to pass the reciprocal best blast hit filter applied, indicates their DNA sequences may have diverged between *Chrysemys* and *Apalone*. Among the most highly connected gene (top hub genes) for each module (Table 4), whose expression profile is highly representative of the module's expression profile (the module's eigengene) (Langfelder & Horvath, 2008), we note genes linked to stress response and epigenetic regulation (*Hmgb1*, *Ndrg1*, *Smca5* and *Piwil4*).

**TABLE 4:** Top hub gene found in each *Chrysemys* and *Apalone* co‐expression module.

Module	Gene symbol	Gene name
*Chrysemys*		
CPI‐1	*Tm35b*	Transmembrane protein 35B
CPI‐2	***Hmgb1***	High mobility group box 1
CPI‐3	*Mat2b*	Methionine adenosyltransferase 2b
CPI‐4	*Ddx17*	DEAD‐box helicase 17
CPI‐5	*Zfhx4*	Zinc finger homeobox protein 4
CPI‐6	***Ndrg1***	N‐myc downstream regulated 1
CPI‐7	*Lrc17*	Leucine rich repeat containing 17
CPI‐8	*Nomo2*	NODAL modulator 2
*Apalone*		
ASP‐1	***Smca5***	SWI/SNF related, matrix associated, Actin dependent regulator of chromatin, subfamily A, member 5
ASP‐2	*Lsm7*	LSM7 homolog, U6 small nuclear RNA and mRNA degradation associated
ASP‐3	*Zn646*	Zinc finger protein 646
ASP‐4	*Lich*	Lipase A, lysosomal acid type
ASP‐5	*Lama4*	Laminin subunit alpha 4
ASP‐6	*Kat1*	Kynurenine aminotransferase 1
ASP‐7	*Metk2*	Methionine adenosyltransferase 2A
ASP‐8	*Lmx1a*	LIM homeobox transcription factor 1 alpha
ASP‐9	***Piwil4***	Piwi like RNA‐mediated gene silencing 4
ASP‐10	*Fyb2*	FYN binding protein 2
ASP‐11	*Jam3*	Junctional adhesion molecule 3
ASP‐12	*Lect2*	Leukocyte cell derived chemotaxin 2

*Note*: Bold italic indicates top hub genes representative of their module's expression profile (module eigengenes) linked to stress response or epigenetic regulation.

## 4 DISCUSSION

Understanding the evolution of vertebrate sex determination is hampered because studies of sex‐specific genome‐wide transcription during gonadal development in lineages with evolutionarily labile sex determination have been restricted to TSD taxa. Here, we present the first ever transcriptomic analysis of sexed embryos of a GSD turtle with sex chromosomes (*Apalone*), incubated at two temperatures that produce only males or females in a TSD counterpart (*Chrysemys*). Our study informs the molecular circuitry changes that accompanied the loss of plastic sex determination in *Apalone*'s lineage (Trionychidae) during the evolution of ZZ/ZW sex chromosomes (Badenhorst et al., 2013) from the ancestral TSD condition (Bista et al., 2021; Sabath et al., 2016) represented by *Chrysemys*. Our data uncovered sex‐specific transcriptional patterns underlying sexual development in *Apalone*, and thermal plasticity in this GSD species at stages corresponding with the TSP in *Chrysemys*, a window of time that is fairly conserved across TSD turtles (thus, likely ancestral), and which encompasses approximately the middle third of development (Valenzuela, 2001, 2008). Importantly, the greatest differences between species were detected in the individual gonads (stages 19–22) compared with mixed tissues (stages 9–15), underscoring the divergence in gonadogenesis separating these turtle lineages. Our results are conservative because subtle patterns in the early developing gonad could be masked by expression of nongonadal tissues.

Several nonmutually exclusive factors may drive the observed thermal plasticity in *Apalone*. First, some thermal sensitivity may be relic from its TSD ancestor, which would be reflected in DEGs with similar expression between *Apalone* and *Chrysemys*, as occurs for the male development gene *Wt1* (Valenzuela, 2008b; Valenzuela et al., 2013). Second, some thermal sensitivity in *Apalone* may have diverged via developmental systems drift (True & Haag, 2001), and by genetic drift, since the costs of transcription in eukaryotes are typically low (Lynch & Marinov, 2015) [although excessive expression of ribosomal genes in *Apalone* appears costly and undergoes dosage compensation as a consequence (Montiel et al., 2022)]. Drift would be reflected in genes with thermosensitive expression but of different pattern between species, such as *Dhh*, a testis differentiation gene upregulated at 31°C across stages in *Chrysemys* and at 26°C in *Apalone* during stages 19–22 in our study. Genes whose thermosensitivity in *Apalone* evolved by drift are probably unimportant for sex determination or compensated for in another way (e.g., during translation), as those costs may be sufficiently high to be visible to selection (Lynch & Marinov, 2015). Third, some thermal sensitivity may be entirely novel in either *Apalone* or *Chrysemys* and have arisen after their lineages split from each other. Lastly, it should be noted that not all transcriptional thermal plasticity may function in sex determination or sexual differentiation, and some may simply be due to the exotherm biology of these reptiles. These differences should be kept in mind when interpreting the discussion below. For instance, thermal plasticity in *Apalone* even surpasses that in *Chrysemys* in the number of DEGs by temperature at early stages and is particularly prominent at stage 12 (Figure 3), which is especially intriguing as it is decoupled from sexual development in this GSD species. Whichever its source, thermal sensitivity harbored by GSD taxa over evolutionary time may serve as raw material for natural selection to act upon during future adaptation, including potential reversals to TSD. Such scenarios may have occurred in other turtle lineages, including the TSD sister to softshell turtles, *Carettochelys insculpta* (Literman et al., 2018; Valenzuela & Adams, 2011), precluding their use as proxy for the TSD pattern ancestral to softshells. Thus, given that all other TSD cryptodiran turtles are equally distant from *Apalone*, *Chrysemys* is as appropriate a proxy as any other TSD cryptodiran (Bista et al., 2021), recognizing that some evolution has accrued in both lineages.

### 4.1 Genome‐wide gene expression patterns diverge by temperature in both *Apalone* and *Chrysemys*

To our knowledge, ours is the first application of trajectory analysis to a time series of genome‐wide developmental transcriptomes, which illuminated broad patterns of gene expression underlying sexual development for both turtle species. The *Chrysemys* trajectories showed significant overall differences between temperatures, as expected given its TSD mechanism, and underscoring the power of this method to capture and quantify hyperdimensional transcriptomic patterns. Notably, 31°C elicited greater change in gene expression in *Chrysemys* than 26°C (trajectories differed in magnitude), providing a molecular explanation for why warmer temperatures in *Chrysemys* and other TSDIa turtles have greater potency to feminize embryos than the masculinizing potency of colder temperatures (Georges, 1989; Valenzuela et al., 2019). By contrast, all trajectories for *Apalone* differed in direction and shape, but never in magnitude, indicating that the total amount of change in gene expression was canalized, and that instead, different genes changed expression by temperature or by sex, and did so in a distinct manner through time in this GSD turtle.

Importantly, the trajectories began diverging between temperatures as early as stage 12 in *Chrysemys* (Figure 2C), indicating that differential expression of sex‐related candidate genes in TSD turtles as early as stages 9 and 12 reflect genome‐wide responses, and supporting the notion that temperatures experienced before the canonical TSP may influence sex ratios to some degree (Czerwinski et al., 2016; Gómez‐Saldarriaga et al., 2016; Radhakrishnan et al., 2017, 2018; Valenzuela, 2001, 2008a, 2008b, 2010; Valenzuela et al., 2006, 2013; Valenzuela & Shikano, 2007). A remarkable difference between species was detected at stage 15, when differential expression accentuated in *Chrysemys* whereas *Apalone* trajectories remained parallel (Figure 2). This is significant, because no broad differences in the sequence or overall timing of gonadogenesis events were detected between *Apalone* and TSD turtles previously (Greenbaum & Carr, 2001), such that our data expose the conspicuous canalization in *Apalone* at the stage that marks the onset of the TSP in *Chrysemys*. Indeed, as transcriptional patterns became more complex in *Chrysemys*, pattern of expression in *Apalone* remained impervious to the interaction effect of sex‐by‐temperature.

The most striking differences among trajectories within and between species occurred during the mid‐ and late‐TSP in *Chrysemys* (Figure 2e,f), also revealing extensive thermal plasticity in genome‐wide transcription in *Apalone*. Specifically, differential gene expression increased in *Chrysemys* and sex‐by‐temperature interactions became more complex in *Apalone*. Intriguingly, the diverging trajectory paths observed in stage 19 gonads in both taxa, lessened at stage 22 in *Chrysemys*, and between male and female *Apalone* under 26°C (Figure 2), whereas they were exacerbated between the sexes at 31°C in *Apalone*. This may suggest that sexually dimorphic genome‐wide expression is less critical by the end of the TSP in the developing gonads of *Chrysemys*, and that the ancestral feminizing effect of warm temperatures (revealed by the greater magnitude of female trajectories in *Chrysemys*—see above) may be counteracted in *Apalone* by exaggerated sexually dimorphic transcription.

Using WGCNA we detected shifts in co‐expression patterns of gene modules between *Apalone* and *Chrysemys* during female and male embryogenesis, some reflecting their distinct mechanisms of sexual development and perhaps driven by positive selection, while others may, in part, be indicative of developmental systems drift (True & Haag, 2001). Overall, the WGCNA analysis uncovered groups of genes within modules that were co‐expressed similarly in both species, but whose relationship to other groups of genes (their correlated expression) was modified between species during their 161 My of independent evolution. The module hub genes identified in this analysis (Table 4) represent new gene candidates of interest for sexual development. Among these, *Hmgb1* is a stress response gene (Yu et al., 2015), and *Ndrg1* is associated with hormone and stress responses and may play a role in follicular development in humans (Nishigaki et al., 2022), whereas *Smca5* and *Piwil4* are involved in epigenetic regulation, and thus, plausible mediators of plasticity.

### 4.2 What genes canalized softshell turtle sexual development?

**FIGURE 5:**
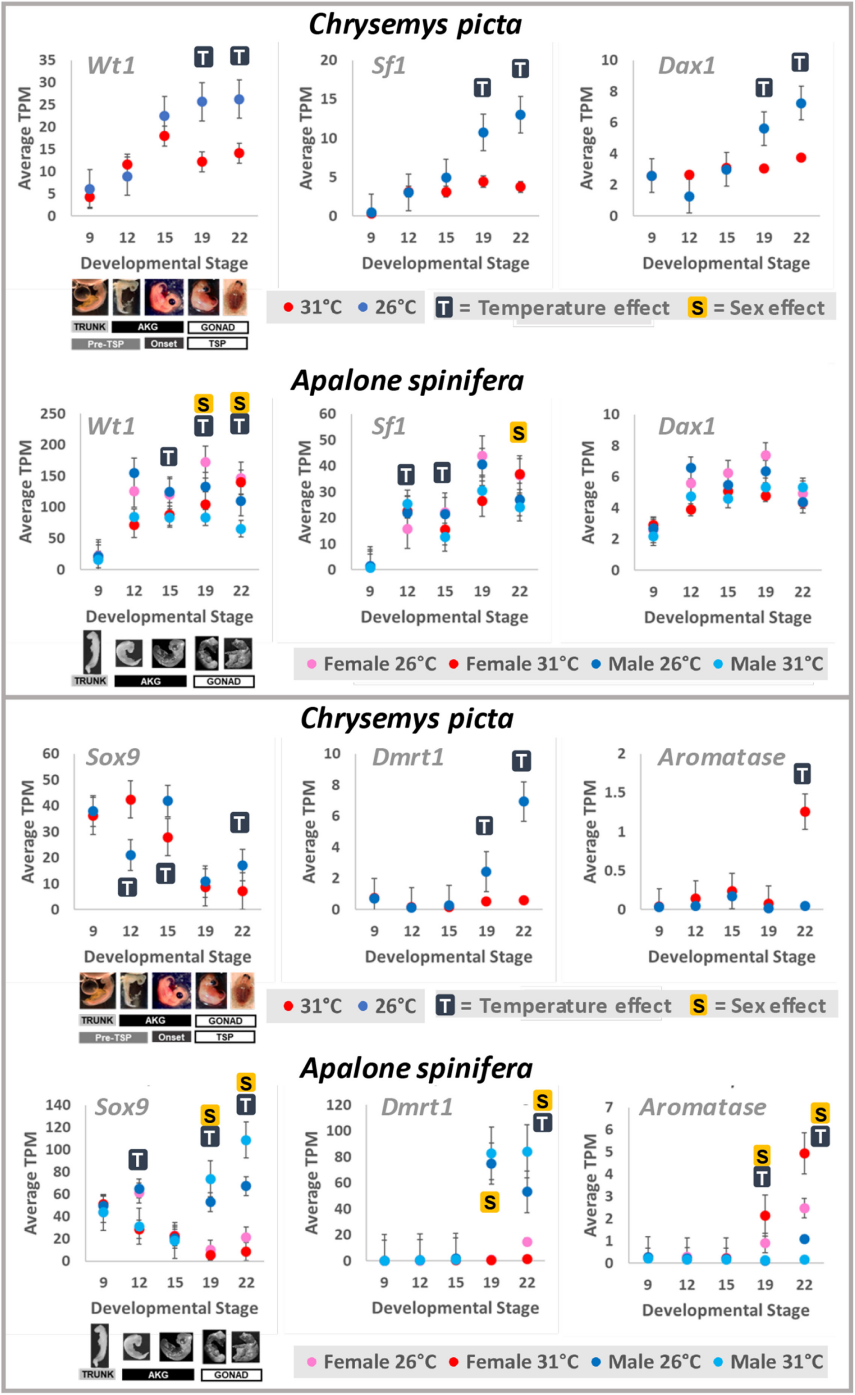
Transcriptional profiles from this study showing six genes of interest in *Chrysemys picta* and *Apalone spinifera*. “S” indicates DEGs between sexes and “T” indicates DEGs between temperatures. We note that the DNA sequence of *Wt1* in the *Chrysemys* genome v3.0.3 is split across two scaffolds, one containing the KTS region [a tripeptide present or absent in two *Wt1* splice variants conserved across vertebrates (Hammes et al., 2001)], and the other containing the upstream part of the gene, such that the reference transcriptome contains transcripts of similar expression corresponding to these two sub‐regions. We report the partial KTS‐containing region here as it is most comparable between species.

Comparing the expression of several gene candidates between species suggest potential mechanistic explanations for the evolution of their contrasting sex determination, which will require future testing. In *Apalone* embryos, *Sox9*, *Aromatase*, and *Dmrt1* all showed sex‐specific expression at stage 19 irrespective of temperature, as did *Sox9* at stage 22 (whereas *Aromatase* and *Dmrt1* only displayed this pattern at 31°C at this stage) (Figure 5), such that these genes were supported as core components of the gonadal differentiation cascade as in other turtles and vertebrates (Capel, 2017; Ge et al., 2017; Morrish & Sinclair, 2002; Radhakrishnan et al., 2017; Smith et al., 2009; Valenzuela, 2008a, 2008b, 2010; Valenzuela et al., 2013; Valenzuela & Shikano, 2007). Interestingly, the upregulation in *Apalone* of *Sox9* (a testis‐development gene) at 31°C compared to 26°C in stage 19 males may have evolved to counter the feminizing effect that 31°C had in their TSD ancestor. By contrast, *Sox9* and *Dmrt1* (another testis‐development gene) were thermo‐insensitive in *Apalone* females at stage 19, perhaps because the signal from the ZW genotype canalizes their transcription. Yet, at stage 22, *Sox9* and *Dmrt1* were upregulated in females at 26°C, the ancestral TSD expression pattern for these male‐development genes. On the contrary, *Aromatase* (a female‐development gene) was upregulated at 31°C in *Apalone* females, the typical pattern observed in *Chrysemys* and other TSD turtles (Czerwinski et al., 2016; Radhakrishnan et al., 2017; Valenzuela & Shikano, 2007). By contrast, *Aromatase* was mostly thermo‐insensitive in *Apalone* males (significantly upregulated but with low fold change at 26°C during stage 22), as if the two Z chromosomes of males are needed to canalize its transcription or to downregulate it below the threshold that would induce ovarian development (Figure 5). The onset of *Aromatase* differential expression occurred earlier in *Apalone* than in *Chrysemys* (Figure 5), similar to the earlier expression of sexually dimorphic traits observed in other GSD vertebrates (Gross et al., 2017; Valenzuela, 2008; Valenzuela et al., 2003), and which might cause (or be the result of) the earlier commitment of gonads of GSD reptiles to their sexual fate than in their TSD close relatives (Greenbaum & Carr, 2001; Neaves et al., 2006). Additionally, *Dmrt1* at stage 19 in *Apalone* showed no thermal plasticity, and the onset of its canalized sex‐specific transcription is conserved with *Chrysemys*, underscoring the *Dmrt1*'s central role in sexual development in turtles. Yet, *Dmrt1* differential expression occurs earlier (in early TSP) in *Trachemys* (Ge et al., 2017) than in *Apalone* and *Chrysemys* (mid‐TSP) [see also (Mizoguchi & Valenzuela, 2020)], suggesting that *Dmrt1* may be more important for male differentiation than for sex determination, consistent also with its autosomal location in *Apalone* and its trionychid relative *Pelodiscus* (Lee et al., 2019).

**FIGURE 6:**
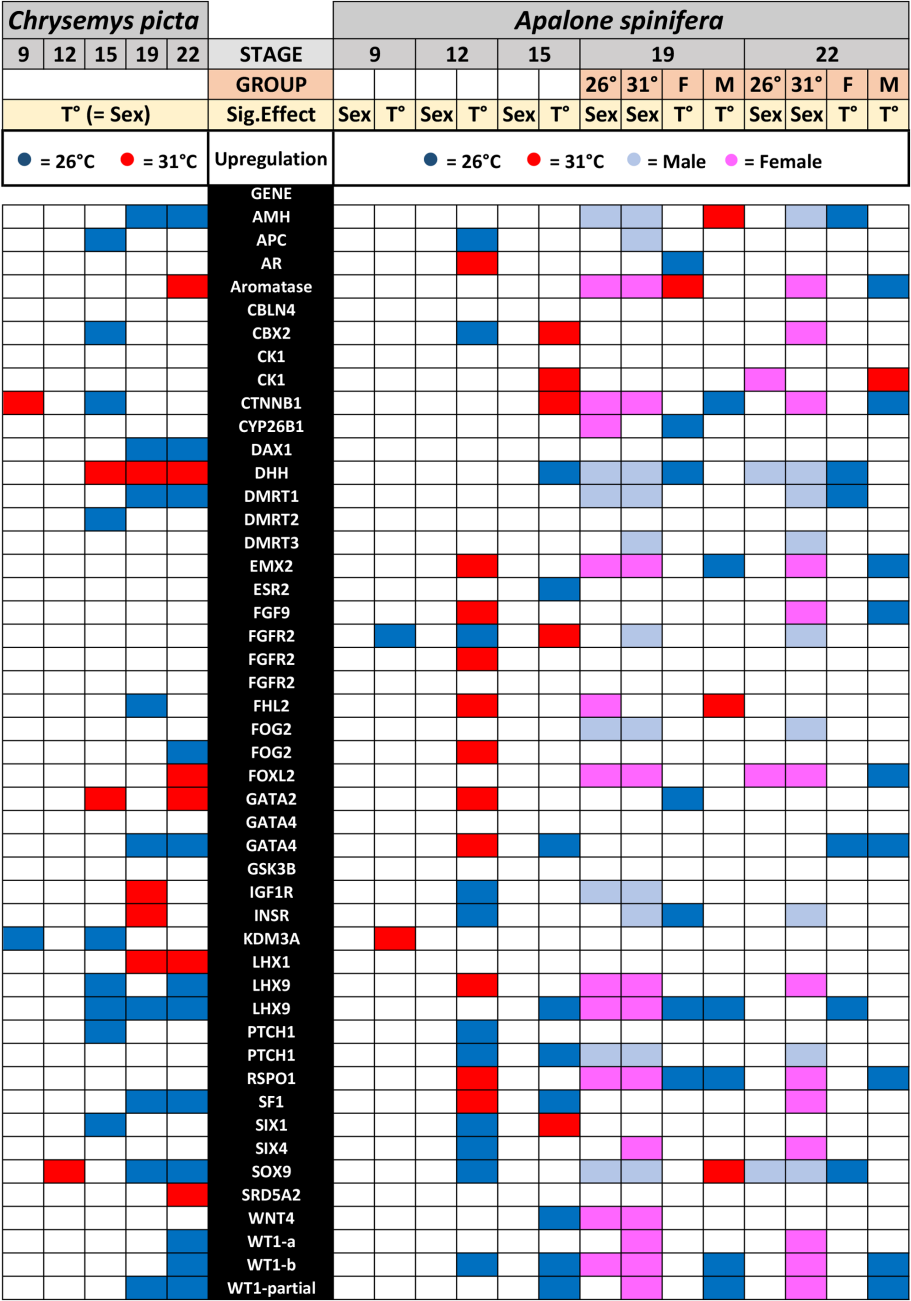
Genes of interest from the vertebrate sexual development network and their expression pattern by sex and/or temperature. First row indicates the developmental **stage** (9–22), followed by the **group** from the factorial design in *Apalone*, i.e., incubation temperature (26°C or 31°C), and sex (F, Female and M, Male). **Sig (significant) effect T** indicates significant difference between temperature treatments, while **Sex** indicates significant difference between males and females. Red = 31°C; darker blue = 26°C; pink = female; light blue = male. Blank cells denote non‐significant effects. WT1‐a, WT1‐b, and WT1‐patial, correspond to protein annotations for isoform sequences X1 and X2 present in the *Chrysemys picta* genome assembly v3.0.3, and the partial Wt1 protein sequences included in Figure 5, respectively (not to the‐KTS, +KTS, and +KTS‐short discussed in the text).

**FIGURE 7:**
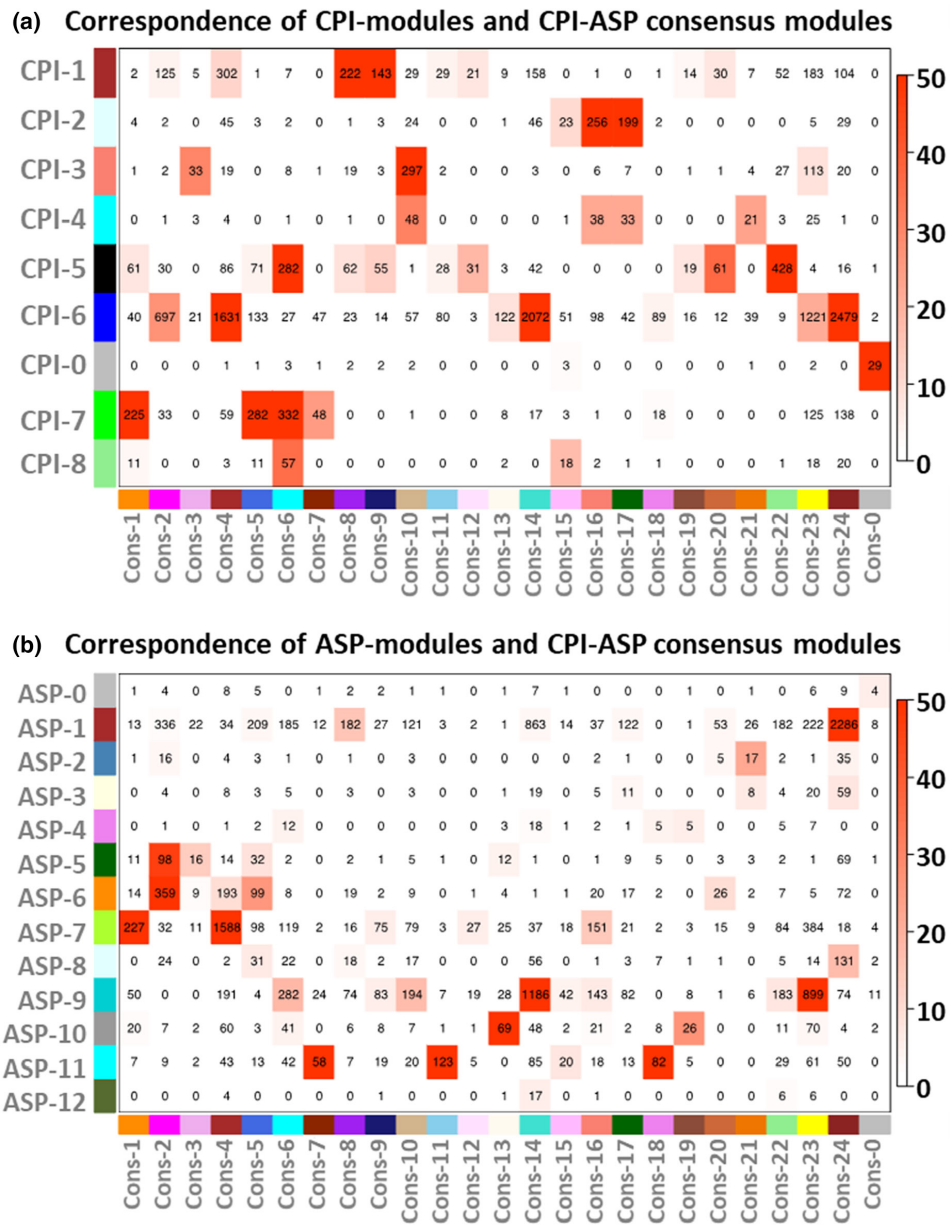
Correspondence of *Chrysemys* (a) and *Apalone* (b) specific modules (genes with highly correlated expression within each species); to consensus modules (genes with highly correlated expression in both species). Numbers within cells indicate the number of genes that overlap between species‐specific and consensus modules. Red scale indicates –log(p) where *p* is the Fisher's exact test *p*‐value, and greater intensity indicates a more significant overlap between modules.

**FIGURE 8:**
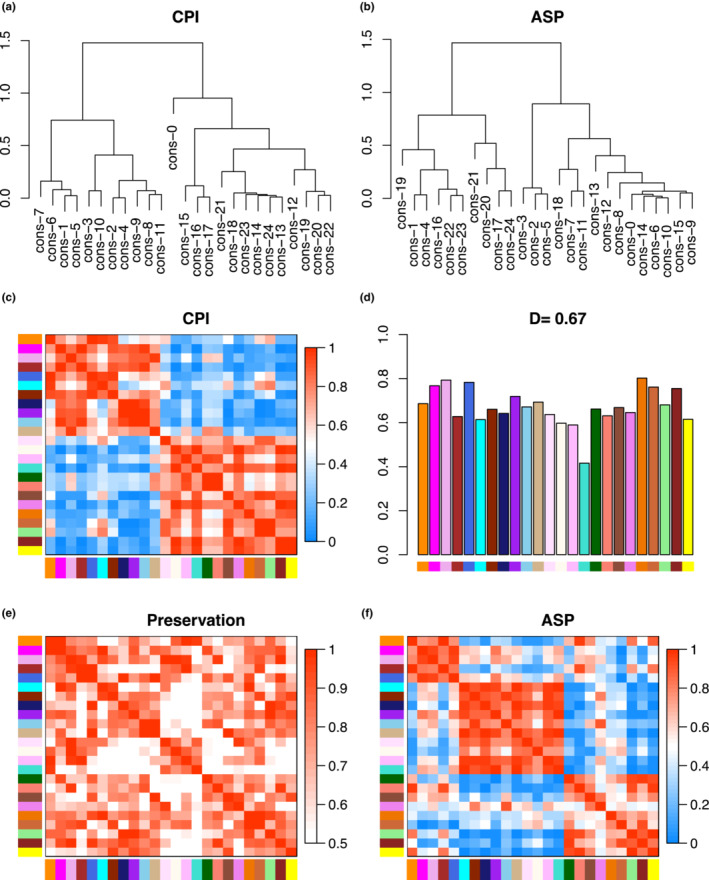
WGCNA results comparing *Chrysemys* and *Apalone* to each other. (a) Clustering for *Chrysemys* and (b) *Apalone* of consensus gene co‐expression modules. Panels (c) (for *Chrysemys*) and (f) (for *Apalone*) contain heatmaps of eigengene networks (interconnectivity plots) which summarize the relationship among module co‐expression patterns within species by clustering their eigengenes (weighted average gene expression profile). Red in heatmaps indicates greater adjacency (greater positive pairwise correlation between consensus modules). (d) Mean preservation of adjacency for all eigengenes which indicates the degree of similarity in module co‐expression correlation patterns. More consistently red columns in panel (e) equate to higher values for each module in panel (d). (e) Preservation of the network between *Chrysemys* and *Apalone*. More intense red indicates greater preservation, and thus greater similarity of the modules between species. Note that module colors in this figure correspond to consensus modules in Figure 7.

Significant changes were detected in the transcription of *Sf1*, *Wt1*, *Gata4*, and *Dax1* in *Apalone* compared with the TSD pattern seen in *Chrysemys*. In humans, *Sox9* works with *Sf1*, *Wt1*, and *Gata4* to regulate *Amh* expression, among which *Sox9* may be most critical (Marshall & Harley, 2000). The expression patterns of these genes observed here in *Chrysemys* agreed with this model, showing upregulation at MPT (26°C) during the TSP. In *Apalone*, however, only *Sox9* and *Amh* are upregulated in males in late development (stages 19 and 22—the mid‐ and late‐TSP of *Chrysemys*), suggesting that the genes with a lesser role no longer cooperate to regulate *Amh*, and supporting *Sox9* as most critical for this function (Marshall & Harley, 2000). In fact, *Wt1* and *Sf1* were upregulated in *Apalone* females at this point, and *Gata4* expression was sexually monomorphic. Also in agreement, *Dax1*, a positive regulator of *Sox9* and inhibitor of *Sf1*, but which may induce testicular development (Ludbrook & Harley, 2004), showed no differential expression by temperature or sex in *Apalone*, suggesting that *Dax1* may no longer regulate these two genes in this GSD turtle. It is worth noting that *Sf1* translocated to the ZW sex chromosomes in *Apalone* (Lee et al., 2019) within a region expanded in the W sex chromosome that was invaded by R2 retrotransposons, which affect the expression of nearby genes (Montiel et al., 2022). But whether *Sf1* took over as a master switch gene remains untested. A hypothesis is that *Sf1's* putative expansion on the W chromosome could have altered how these genes regulate *Amh* during the TSP‐equivalent stages, particularly because *Sf1* was not upregulated at 26°C in *Apalone* as it is in *Chrysemys*, and *Sf1* is a downstream target of *Wt1*, which was upregulated at 26°C during stages 19 and 22 (as was *Gata4* at stage 22). This would render *Wt1*'s thermosensitivity moot for sex determination. We note that *Wt1* tended to be upregulated at 26°C during stages 12–15 in *Apalone spinifera* as in its congener *A. mutica* (Valenzuela, 2008b), and within males at stages 19–22 (though expression in females was even higher at these two stages). Such upregulation at cool temperatures matches the pattern in *Chrysemys* and thus appears relic in the softshell turtle lineage (Valenzuela, 2008b). But the significance of upregulation of *Wt1* in females observed at stages 19–22 in *Apalone* is obscure given the clear role of *Wt1* for testicular development in vertebrates and the lack of *Wt1* upregulation in females across disparate taxonomic orders (Morrish & Sinclair, 2002; Valenzuela et al., 2013). Perhaps *Wt1* regulatory role was lost in *Apalone* or shifted to female‐development, a major potential evolutionary overhaul that warrants further investigation.

Notably, given the level of thermosensitive transcription observed in *Apalone*, a counter‐mechanism (presumably governed by the sex chromosomes) must exist to prevent sex ratios from being altered by temperature, which was confirmed by incubation experiments (Bull & Vogt, 1979; Ewert & Nelson, 1991). Consistently, very few genes exhibited both sex‐specific thermo‐insensitive expression in *Apalone* and differential expression by temperature in *Chrysemys* early in development (stages 9–15). Of the genes that lost thermosensitive expression in *Apalone* compared with *Chrysemys*, *Sc5a1*, *Msi1h*, and *Ppil2* are located on the sex chromosomes in *Apalone* (Bista et al., 2021). Furthermore, *Sc5a1* is a sodium‐dependent glucose transporter (Turk et al., 1994), which may be relevant given the potential for calcium to help regulate TSD (Castelli et al., 2020). Furthermore, *Rrp8* is an intriguing candidate upregulated in *Apalone* males at stage 9, involved in chromatin remodeling (He et al., 2019) and ribosomal DNA silencing (Murayama et al., 2008), which is relevant given the contrasting epigenetic machinery transcription between *Chrysemys* and *Apalone* (Radhakrishnan et al., 2018).

In *Pelodiscus sinensis*, the Chinese softshell turtle that shares a homologous sex chromosome system with *Apalone* (Badenhorst et al., 2013; Rovatsos et al., 2017), experimental overexpression of *Amh* masculinized female embryos while its silencing feminized male embryos (Zhou et al., 2019). *Amh* was upregulated in *Apalone* males at stage 19 (irrespective of temperature) and at stage 22 under 31°C (Fig. 6), perhaps countering the feminizing effect of warm temperatures and thus having a male‐canalizing effect. The additional 31°C‐biased thermosensitive *Amh* expression at stage 19 observed in males may help masculinize ZZ individuals incubated at ancestrally feminizing temperatures. Meanwhile, the loss of detectable thermosensitivity of *Amh* in males at stage 22 suggests a key role of *Amh* in male differentiation via thermally canalized transcription (not in sex determination, given *Amh's* late expression, later than in *Pelodiscus*). Furthermore, *Amh's* autosomal location (Bista et al., 2021) rules it out as a master sex‐determining gene in *Apalone*. On the contrary, the ancestrally masculinizing effect of *Amh* upregulation at 26°C in females at stage 22 appears to be overridden by a feminizing factor(s) in genotypic females (we hypothesize that this may be accomplished by a W‐linked factor, or a dose‐dependent Z‐linked factor).

Thus, our results suggest that despite the evolution of sex chromosomes in *Apalone*'s lineage, embryos need to counter the effects of relic thermosensitivity for proper sexual development, revealing a more complex interplay between residual (and novel) thermosensitivity (i.e., transcriptional plasticity) and genotypic sex determination (i.e., developmental canalization) than previously anticipated. It is interesting that we observe this pattern particularly in genes regulating the development of males (*Amh*, *Sox9*, *Dmrt1*), who are the homogametic (ZZ) sex in this species (Badenhorst et al., 2013). This suggests that two Z chromosomes may not suffice for male sex determination in *Apalone*, as observed in birds (Smith et al., 2009), and leads to the hypothesis that the W chromosome contains a/the sex‐determining factor in *Apalone* rather than sex being determined by a Z‐linked dosage system.

### 4.3 New and old candidates for sex determination emerge, informing models of temperature‐dependent sex determination

We also compared our results to recent models of TSD. In *Trachemys scripta* (Weber et al., 2020) (referred to as *Trachemys* hereafter), an increase in calcium at FPT (possibly linked to TRP proteins) causes phosphorylation of STAT3 which binds to *Kdm6b* (a positive regulator of *Dmrt1*), inhibiting its expression and that of *Dmrt1*. Importantly, protein activity of TRPV4 and phosphorylation status of STAT3 appear more critical than transcription and protein levels (Weber et al., 2020). Consistently, *Trpv4* expression in *Chrysemys* was monomorphic in our study, such that TRPV4 may play a sentinel role with monomorphic transcription adequate to respond to the environmental cue, as observed in other systems (Mateus et al., 2014). Unlike in *Trachemys* (Weber et al., 2020), *Stat3* in *Chrysemys* was upregulated at FPT (marginally at stages 9 and 15, and significantly at stage 19), whereas *Kdm6b* was upregulated at MPT at stages 9 and 19 but at FPT at stage 15, instead of steadily at MPT as in *Trachemys* (Ge et al., 2018). This suggests that KDM6B may be a less important regulator of *Dmrt1* in *Chrysemys*, consistent with *Dmrt1*'s strong upregulation in males during mid‐ and late TSP [this study and qPCR analyses (Mizoguchi & Valenzuela, 2020)], indicating that *Dmrt1* plays a role in male sex differentiation but not sex determination in *Chrysemys*, counter to that proposed for *Trachemys* (Ge et al., 2017). Together, these observations strongly support the hypothesis that developmental systems drift has occurred between these two closely related emydids (Mizoguchi & Valenzuela, 2020).

Of the stress response genes potentially involved in TSD (Straková et al., 2020), only *Atp2a2* was male‐upregulated across stages 9–15 in *Apalone* (Table S15). But other genes were upregulated at 26°C in stage 19–22 gonads, including *Atp2a2* and *Ano1* [both implicated in calcium transport or levels (Lytton & Maclennan, 1988; Yang et al., 2008)], genes linked to the regulation of steroids and hormones and implicated in sexual development [*Ppargc1a*, *Sst*, *Tgfb1i1* (Fujimoto et al., 1999; Patel, 1999; Tcherepanova et al., 2000)], and *Fosl2* which is involved in the formation of AP‐1 (Hess et al., 2004), a transcription factor complex that interacts with SF1 (NR5A1) (Dubé et al., 2009). Interestingly, all stress‐related DEGs in *Chrysemys* were both differentially expressed by sex and by temperature at some stage in *Apalone*.

Another candidate, *Cirbp*, was proposed as an activator of STAT3 given its connection to calcium and TRPV4 signaling (Weber et al., 2020). *Cirbp* shows temperature‐dependent allele specific expression correlated with sex ratios in *Chelydra serpentina* (Schroeder et al., 2016), where one of two alleles is thermosensitive, and its expression is correlated with female‐biased clutches. Here, we also observe upregulation at FPT of two *Cirbp* isoforms across stages in *Chrysemys* (a third rare isoform exhibited monomorphic expression). Intriguingly, three *Cirbp* isoforms were observed in *Apalone* and lacked sex‐specific expression but were always upregulated at 31°C in both sexes, perhaps reflecting a stress response (Liao et al., 2017) to constant warm temperatures but with no role on sexual development in *Apalone*, as may also occur in *Chrysemys*. It should be noted that *Cirbp*'s association with femaleness remains tenuous, because the expression of the allele associated with male‐biased clutches in *C. serpentina* (Schroeder et al., 2016) was not thermosensitive, rather carrying that allele was associated with maleness, whereas carrying the alternate allele was associated both with temperature and femaleness, such that its role could not be detangled in that study.

The model by Weber and collaborators (Weber et al., 2020) fits well with the CaRe (cellular Calcium and Redox) status hypothesis (Castelli et al., 2020), and proposes that pathways related to stress and calcium signaling could be co‐opted for the evolution of TSD. Here, we detected monomorphic expression of many stress‐related genes in both species, ruling them out for a dual role in sexual development. Interestingly, data from later stages in *Apalone* (and not *Chrysemys*) suggest a possible relationship between sex and stress, but experiments detangling sex and temperature are needed to test whether the same may occur in *Chrysemys*. *Atp2a2* is particularly intriguing because it is related to calcium transport (Lytton & Maclennan, 1988), was a DEG at stages 15–19 in *Chrysemys* (onset and mid TSP), and at least one transcript was differentially expressed by sex at every stage in *Apalone* and by temperature for stages 9, 12, and 19. Finding stress‐response genes with sustained upregulation at warmer temperatures in *Chrysemys* is particularly important to elucidate the molecular architecture underlying increased feminization and mortality predicted for this and many TSD turtles under climate change (Jensen et al., 2020; Valenzuela et al., 2019).

## 5 CONCLUSIONS

Our unprecedented trajectory analysis of transcriptomic time series from sexed embryos of a GSD reptile incubated under temperatures that induce maleness and femaleness ancestrally, illuminated the evolution of sexual development in a turtle with sex chromosomes. We found that extensive plasticity in transcription persists over millions of years after developmental canalization evolves, eliciting active transcriptional countermeasures to prevent phenotypic and genotypic mismatch. Our findings inform our understanding of how environmental cues might be translated into molecular signals for development by (a) identifying new and validating well‐known members of the vertebrate sexual development network, (b) identifying novel sex‐determining candidate genes in a ZZ/ZW turtle, (c) strengthening the hypothesis that stress and sexual development might be associated, and (d) highlighting evolutionary remodeling of transcriptional patterns that accompanied the transition from plasticity to canalization.

Several inferences follow our results:

1. The evolution of canalization does not require genome‐wide environmental insensitivity.
2. Lingering thermosensitivity may be neutral to natural selection and co‐optable for the evolutionary reversal from GSD to TSD, or for other thermal adaptation.
3. Climate change may alter gene expression in GSD turtles (not just TSD taxa), potentially triggering increased canalization in sexual development (i.e., genetic assimilation) or a turnover of sex determination.

## AUTHOR CONTRIBUTIONS

**Thea B Gessler:** Data curation (lead); formal analysis (lead); investigation (lead); writing – original draft (equal); writing – review and editing (equal). **Zhiqiang Wu:** Data curation (supporting); formal analysis (supporting); investigation (equal); writing – review and editing (supporting). **Nicole Valenzuela:** Conceptualization (lead); funding acquisition (lead); investigation (supporting); project administration (lead); supervision (lead); writing – original draft (supporting); writing – review and editing (equal).

## FUNDING INFORMATION

This work was funded in part by NSF grants MCB 1244355 and IOS 1555999 to N.V. Open access funding provided by the Iowa State University Library.

## BENEFIT SHARING

Research benefits will result from the sharing of these datasets on public databases as described in the Data Accessibility section.

## Supporting information


**Data S1.** Supporting Information

## Data Availability

All sequencing reads and gene expression data are deposited in the Short Read Archive at NCBI: BioProjects PRJNA683586 (*Apalone spinifera* embryos—SRR13224849‐SRR13224888) and PRJNA594037 (*Chrysemys pict*a embryos—study number SRP237291; SRR10674595‐SRR10674614). *Apalone spinifera* genome assembly is available at NCBI (BioProject: PRJNA837702).
